# Comparison of primary target volumes delineated on four-dimensional CT and ^18^ F-FDG PET/CT of non-small-cell lung cancer

**DOI:** 10.1186/1748-717X-9-182

**Published:** 2014-08-15

**Authors:** Yi-Li Duan, Jian-Bin Li, Ying-Jie Zhang, Wei Wang, Feng-Xiang Li, Xiao-Rong Sun, Yan-Luan Guo, Dong-Ping Shang

**Affiliations:** Department of Radiation Oncology, Shandong Cancer Hospital and Institute, Jinan, 250117 China; PET/CT Room, Shandong Cancer Hospital and Institute, Jinan, 250117 China; Big Bore CT Room, Shandong Cancer Hospital and Institute, Jinan, 250117 China; Medicine and life sciences college of Shandong Academy of Medical Sciences, Jinan University, Jinan, Shandong Province 250200 People’s Republic of China

**Keywords:** Non-small cell lung cancer, Fluorodeoxyglucose positron emission tomography, Four-dimensional computed tomography, Standardized uptake value

## Abstract

**Background:**

To determine the optimal threshold of ^18^ F-fluorodexyglucose (^18^ F-FDG) positron emission tomography CT (PET/CT) images that generates the best volumetric match to internal gross target volume (IGTV) based on four-dimensional CT (4DCT) images.

**Methods:**

Twenty patients with non-small cell lung cancer (NSCLC) underwent enhanced three-dimensional CT (3DCT) scan followed by enhanced 4DCT scan of the thorax under normal free breathing with the administration of intravenous contrast agents. A total of 100 ml of ioversol was injected intravenously, 2 ml/s for 3DCT and 1 ml/s for 4DCT. Then ^18^ F-FDG PET/CT scan was performed based on the same positioning parameters (the same immobilization devices and identical position verified by laser localizer as well as skin marks). Gross target volumes (GTVs) of the primary tumor were contoured on the ten phases images of 4DCT to generate IGTV_10_. GTV_PET_ were determined with eight different threshold using an auto-contouring function. The differences in the position, volume, concordance index (CI) and degree of inclusion (DI) of the targets between GTV_PET_ and IGTV_10_ were compared.

**Results:**

The images from seventeen patients were suitable for further analysis. Significant differences between the centric coordinate positions of GTV_PET_ (excluding GTV_PET15%_) and IGTV_10_ were observed only in z axes (P < 0.05). GTV_PET15%_, GTV_PET25%_ and GTV_PET2.0_ were not statistically different from IGTV_10_ (P < 0.05). GTV_PET15%_ approximated closely to IGTV_10_ with median percentage volume changes of 4.86%. The best CI was between IGTV_10_ and GTV_PET15%_ (0.57). The best DI of IGTV_10_ in GTV_PET_ was IGTV_10_ in GTV_PET15%_ (0.80).

**Conclusion:**

None of the PET-based contours had both close spatial and volumetric approximation to the 4DCT IGTV_10_. At present 3D-PET/CT should not be used for IGTV generation.

## Background

Worldwide, lung cancer is the most common cause of cancer-related mortality [1]. About 80% of the cases of lung cancer are non-small-cell lung cancer (NSCLC) [2]. Radiotherapy plays a major role in the management of patients with NSCLC who cannot tolerate or refuse surgery. Unfortunately, the prognosis of patients with NSCLC remains poor because of high rates of local failure and distant metastases [3, 4], with local control rates of approximately 50% after radical radiotherapy [5]. A geometric target miss induced by tumor motion during radiotherapy is considered as one of the main reasons for local failure [6]. In order to account for geometric uncertainties due to internal variations in tumor position, size, and shape, the International Commission on Radiation Units and Measurements (ICRU) report 62 introduced the concept of an internal target volume (ITV) [7]. For lung tumors, respiratory motion is the major consideration for the ITV.

Currently, four-dimensional CT (4DCT) is widely used for the simulation of lung cancer. It is a reliable and effective tool for assessing tumor and organ motion [8, 9] and can provide patient-specific information about tumor position, shape, and size at different phases of the respiratory cycle. The internal gross target volume (IGTV) can make the determination of the ITV more efficient [6]. IGTV_10_ is generated by combining all 10 individual gross tumor volumes (GTVs) contoured in each phase of the 4DCT dataset, which is thought to encompasses the motion information for the tumor in the whole respiratory cycle [10]. Therefore, it is expected to provide the most accurate IGTV based on a given 4DCT dataset [11].

As a functional imaging modality, ^18^ F-fluorodeoxyglucose (^18^ F-FDG) positron emission tomography/computer tomography (PET/CT) images have been shown to have greater specificity and sensitivity than CT alone for the diagnosis and staging of NSCLC patients [12]. Furthermore, the interobserver variability, as well as the intraobserver variability, could be significantly reduced when the ^18^ F-FDG PET image was used for tumor volume delineation [13, 14]. In addition, since three-dimensional PET (3D-PET) images are acquired over several minutes and represent the accumulated traces of multiple respiratory cycles, they may be capable of accounting for movement by indicating the average location of a tumor over time [15]. A phantom study by Caldwell et al. [16] concluded that PET imaging could more accurately depict the 3D volume of a moving phantom compared with spiral CT. Therefore, 3D-PET/CT might represent the ITV of a tumor. However, the optimal threshold values for patients with NSCLC have never been reported.

Hence, We perform this study to investigate the appropriateness of the threshold method to determine the best volumetric match to 4DCT-based IGTV_10_ when contouring the primary tumor volume of NSCLC. Additionally, the feasibility of 3D-PET/CT images was evaluated with respect to incorporating tumor motion into the radiation target for NSCLC.

## Methods

### Patients

This study was approved by the Shandong Cancer Hospital and Institute review board, and 20 patients provided written informed consent. Patients with histologically proven primary NSCLC who were scheduled to undergo radiotherapy were eligible for this study, excluding those with atelectasis and/or obstructive pneumonia. None of them had previously been treated with radiotherapy or chemotherapy for their lung tumor. Between December 2012 and December 2013, 20 patients were enrolled in this study. The maximal standardized uptake value (SUV_max_) in the tumor of 3 patients was 2.89, 3.16 and 3.27, respectively. Therefore, they were not suitable for further analysis as several of the threshold-based contouring methods used would not discriminate between the tumor and background lung uptake. The other 17 patients included 13 men and 4 women, with a median age of 66 years (range, 45–84 years). Six patients had centrally located lesions, and eleven patients had peripherally located lesions. The median of the SUV_max_ for the primary tumors was 11.34 (range from 6.07 to 25.51). Table 1 summarized the characteristics of the 17 patients and their primary tumors.

**Table 1:** **Baseline characteristics of patients and the maximal standardized uptake values of every primary tumor**

Patients (n)	Sex	Age (y)	Tumor location	Histology	Tumor stage	SUV _max_	Tumor size* (mm)	Tumor volume ^+^ (cm ^3^)
1	M	66	Upper lobe	Adenocarcinoma	T2N2	25.51	36	17.49
2	F	68	Lower lobe	Adenosquamous carcinoma	T2N2	11	46	139.72
3	M	70	Upper lobe	Adenocarcinoma	T2N2	7.71	18	4.81
4	M	79	Upper lobe	Adenocarcinoma	T1N2	8.8	33	26.40
5	F	49	Lower lobe	Squamous cell carcinoma	T2N2	8.85	38	38.59
6	M	66	Upper lobe	Squamous cell carcinoma	T2N2	14.41	33	16.28
7	M	75	Upper lobe	Squamous cell carcinoma	T2N2	12.39	32	14.37
8	F	65	Upper lobe	Adenocarcinoma	T2N2	13.41	25	8.12
9	M	76	Lower lobe	Squamous cell carcinoma	T3N2	15.13	52	115.42
10	M	84	Lower lobe	Adenocarcinoma	T2N2	24.81	40	27.53
11	M	68	Upper lobe	Squamous cell carcinoma	T2N2	9.83	40	45.97
12	M	65	Upper lobe	Adenocarcinoma	T2N3	14.38	42	39.43
13	F	67	Upper lobe	Adenocarcinoma	T1N3	6.99	15	5.96
14	M	65	Upper lobe	Squamous cell carcinoma	T4N2	14.52	80	157.67
15	M	45	Upper lobe	Adenocarcinoma	T1N3	6.09	10	5.06
16	M	65	Lower lobe	Adenocarcinoma	T1N2	6.07	24	4.53
17	M	60	Upper lobe	Squamous cell carcinoma	T4N1	11.34	34	19.95

SUV_max_, maximal standardized uptake value; *Tumor sizes represent the major axis of the tumor; ^+^Tumor volume is the average volume of the 10 phases of the gross target volume delineated on four-dimensional CT.

### CT simulation and image acquisition

During the simulation, all patients were immobilized using thermoplastic mask for covering the head, neck and shoulders in the supine position. For each patient, an axial enhanced 3DCT scan of the thoracic region was performed followed by a enhanced 4DCT scan under uncoached free breathing conditions on a 16-slice CT scanner (Philips Brilliance Bores CT) with the administration of intravenous contrast agents. A total of 100 ml of ioversol was injected intravenously, 2 ml/s for 3DCT and 1 ml/s for 4DCT. Details of 3DCT and 4DCT scan as well as image acquisition were given in Li et al. [17]. Then, 3DCT and 4DCT images were transferred to MIM (MIM-6.0.4, MIM Software Inc, Cleveland, OH) imaging software.

### PET/CT simulation and image acquisition

On the same day as the 4DCT scan, the FDG-PET/CT scans of the chest were performed with a integrated PET/CT scanner (Philips Gemini TF Big Bore). Through the same immobilization devices, the patient’s position was identical to that for the 4DCT scan. Two radiation therapists were present to ensure the accuracy of the set-up by laser localizer and skin marks. All patients fasted for at least 6 h before the PET/CT examination. All patients were injected with 7.4 MBq/kg body weight of ^18^FDG and then rested for about 1 h in a quiet room before imaging. The 16-slice CT component was operated with an X-ray tube voltage peak of 120 kV, 90 mA, a slice thickness of 5 mm and an interval of 4 mm, and was used both for attenuation correction of PET data and for localization of FDG uptake in PET images. No CT contrast agent was administered. PET scanning was performed covering the same axial range for 2 min per bed position (total of 3–5 bed positions). Both PET and CT acquisition was performed during free breathing. Data were reconstructed using an ordered subset expectation maximization (OSEM) algorithm and attenuation correction derived from CT data. Then, the PET/CT images were transferred to MIM software.

### Image registration

An initial automatic rigid registration was performed using MIM software. Due to the 3DCT and 4DCT images for the same person were produced during the same imaging session, MIM would consider the images as being registered with each other. After the 3DCT and PET image datasets were co-registered with the help of the transmission CT from PET/CT, the 4DCT images would be auto-registered with the CT component of PET/CT. The registration was then manually adjusted by one radiotherapist, experienced in registering PET/CT images, by matching bony anatomy such as the vertebral bodies at the level of the visible lung lesion. Hence, each contour was transferred to the 3DCT to calculate their specific parameters.

### Target volume delineation

Our investigation focuses on the primary tumors. If the positive lymph nodes could not be separated from the primary tumor visually, they were delineated together as if they were part of the primary tumor. Patients were treated according to the 4DCT-based volumes and PET/CT contours were only used as part of a virtual planning study. Using the lung window setting (W = 1,600, C = −600) and mediastinal window settings (W = 400, C = 40) for the interface if the tumor was close to the mediastinum or chest wall [18], GTVs were manually contoured on all 10 phases of the 4DCT scan by a single radiation oncologist and verified by another radiation oncologist. Both of them did not know the PET results in an effort to decrease bias. IGTV_10_ were derived from the 10 phases of the GTVs. PET/CT-based GTV of the primary tumor (GTV_PET_) was defined by the auto-contouring function of MIM. After identification the primary tumor as a region of interest (ROI), MIM automatically calculated the SUV_max_ of ROI. Eight different threshold methods were used in this study: (1) SUV of 2.0 or greater (SUV2.0); (2) SUV of 2.5 or greater (SUV2.5); (3) 15% of SUV_max_ within the ROI (SUV15%); (4) 20% of SUV_max_ within the ROI (SUV20%); (5) 25% of SUV_max_ within the ROI (SUV25%); (6) 30% of SUV_max_ within the ROI (SUV30%); (7) 35% of SUV_max_ within the ROI (SUV35%); (8) 40% of SUV_max_ within the ROI (SUV40%). All the noncancerous regions within the GTV_PET_, including the areas overlaid by the heart, bone and great vessels, were corrected to exclude manually with the help of the CT of component of PET/CT.

### Volumes comparison

The differences in the position, size, concordance index (CI) and degree of inclusion (DI) between the GTV_PET_ and the IGTV_10_ were compared.

Target volume positions were defined by center of target coordinates and expressed using the x (left-right, LR), y (anterior-posterior, AP) and z (cranial-caudal, CC) coordinates of the center of mass. Centroid shifts in the 3D directions were calculated according to the formula as follows:


The concordance index of volume A and B [CI (A, B)] was defined as the ratio of the intersection of A with B to the union of A and B [19]. The maximum value of CI is 1 if the two volumes are identical, and the minimum value is 0 if the volumes are completely nonoverlapping. That is,


The definition of DI of volume A in volume B [DI (A in B)] is the percentage of the overlap between volume A and B in volume A [20]. The formula is as follows:


The DI can represent the percentage of one volume included by another volume, and 1-DI can represent the percentage of one volume not included by another volume.

### Statistical analysis

Statistical analysis was performed using the SPSS software package (SPSS 17.0). The one-way ANOVA test was used to determine the variations in the DIs of GTV_PET_ and IGTV_10_, and in the CIs of GTV_PET_ and IGTV_10_. The Wilcoxon test was performed to estimate the differences of centroid coordinate positions between GTV_PET_ and IGTV_10_, and also used to estimate the variabilities of target volumes between GTV_PET_ and IGTV_10_. We used the Spearman correlation test to analyze for associations between centroid shifts in the 3D directions and CIs. Values of P < 0.05 were regarded as significant for all the tests. Descriptive statistics were used as appropriate.

## Results

For lesion 15 and 16, the SUV_max_ in the tumor was 6.09 and 6.07, we could not obtain GTV_PET15%_ target volumes as the volume obtained from the SUV15% contours were indistinguishable from background lung activity.

### Centroid shifts of the volumes derived from PET/CT and 4DCT

Table 2 showed the centroid shifts in 3D directions of the IGTV_10_ volumes and the PET/CT volumes. The variations in the centroid coordinate positions in the CC direction of GTV_PET20%_ and IGTV_10_, GTV_PET25%_ and IGTV_10_, GTV_PET30%_ and IGTV_10_, GTV_PET35%_ and IGTV_10_, GTV_PET40%_ and IGTV_10_, GTV_PET2.0_ and IGTV_10_, GTV_PET2.5_ and IGTV_10_ were significant (z = −2.131, −2.131, −2.012, −2.012, −2.012, −2.012, −2.226; P = 0.033, 0.033, 0.044, 0.044, 0.044, 0.044, 0.026), while in the LR and AP directions were not significant (P > 0.05). The variations in the LR, AP and CC directions of GTV_PET15%_ and IGTV_10_ were not significant (z = −0.502, −0.881, −1.505; P = 0.615, 0.378, 0.132).

**Table 2:** **The centroid shifts of the GTV**_**PET**_ **and IGTV**_**10**_ **in 3D directions (cm)**

Lesion	GTV _PET15%_	GTV _PET20%_	GTV _PET25%_	GTV _PET30%_	GTV _PET35%_	GTV _PET40%_	GTV _PET2.0_	GTV _PET2.5_
1	1.10	1.07	1.04	1.09	1.08	1.07	1.08	1.07
2	0.67	0.75	0.85	0.94	1.05	1.14	0.68	0.79
3	0.77	0.75	0.77	0.74	0.70	0.73	0.78	0.72
4	0.33	0.34	0.37	0.38	0.37	0.35	0.35	0.38
5	0.38	0.28	0.23	0.23	0.25	0.22	0.28	0.23
6	0.09	0.16	0.18	0.18	0.20	0.20	0.12	0.14
7	1.13	1.29	1.36	1.30	1.43	1.55	1.16	1.30
8	0.76	0.75	0.76	0.75	0.79	0.77	0.75	0.73
9	0.22	0.36	0.48	0.54	0.59	0.63	0.14	0.24
10	0.33	0.29	0.33	0.32	0.34	0.35	0.12	0.23
11	0.20	0.32	0.36	0.43	0.46	0.47	0.31	0.37
12	1.16	1.14	1.14	1.11	1.13	1.17	1.17	1.14
13	0.32	0.36	0.41	0.48	0.49	0.50	0.44	0.48
14	1.01	1.02	1.11	1.17	1.29	1.52	0.96	0.98
15	-	0.15	0.17	0.25	0.26	0.22	0.18	0.42
16	-	2.01	1.95	2.02	2.01	2.01	2.01	1.98
17	0.43	0.38	0.43	0.44	0.50	0.52	0.33	0.39
Median	0.43	0.38	0.48	0.54	0.59	0.63	0.44	0.48
sd	0.37	0.50	0.49	0.49	0.50	0.54	0.51	0.49

sd, standard deviation.

### Volume variation

**Figure 1:**
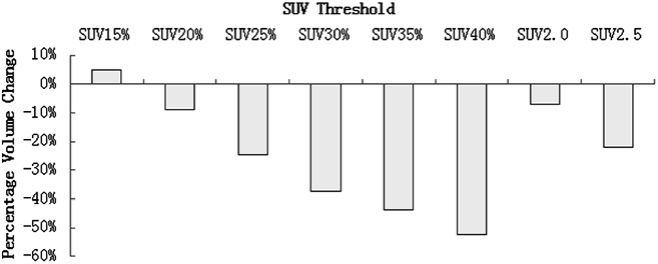
**Median percentage volume change of GTV**_**PET**_ **to IGTV**_**10**_ **.** SUV n = SUV of n; SUV n% = n% of maximum SUV.

The volumes of primary tumors measured by PET/CT and 4DCT were shown in Table 3. Compared to IGTV_10_, GTV_PET15%_, GTV_PET20%_ and GTV_PET2.0_ showed no significant difference (*P* values were 0.281, 0.102 and 0.687, respectively). Figure 1 illustrated the median percentage of volume changes from GTV_PET_ to IGTV_10_ standardized to the IGTV_10_ for each case. The SUV 15% contour approximated most closely to the IGTV_10_ with the lowest median percentage volume changes of 4.86%. The corresponding values with respect to the IGTV_10_ for the SUV20%, SUV25%, SUV30%, SUV35%, SUV40%, SUV2.0 and SUV2.5 contours were −8.87%, −24.51%, −37.51%, −43.57%, −52.24%, −7.03% and −21.93%, respectively.

**Table 3:** **The volumes of primary tumors measured by PET/CT and 4DCT (cm** ^**3**^ **)**

Lesion	GTV _PET15%_	GTV _PET20%_	GTV _PET25%_	GTV _PET30%_	GTV _PET35%_	GTV _PET40%_	GTV _PET2.0_	GTV _PET2.5_	IGTV _10_
1	24.98	20.59	17	15.31	13.83	11.94	32.75	30.37	24.5
2	159.69	122.17	95.89	72.35	54.9	40.9	135.47	107.23	173.72
3	16.34	10.47	7.82	6.13	4.15	3.59	7.67	5.04	5.9
4	39.49	34.32	29.54	26.89	23.72	21.54	31.11	28	37.66
5	70.9	53.99	46.02	41.27	36.48	32.39	49.45	42.45	44.01
6	19.41	14.44	11.4	9.04	8.07	7.28	20.46	16.81	22.31
7	19.68	13	8.5	5.93	4.27	3.52	18.07	12.76	19.99
8	13.03	10.16	7.9	6.49	5.53	4.68	13.03	10.70	9.8
9	183.15	140.37	115.35	98.64	86.46	76.09	201.56	166.58	152.8
10	24.24	19.76	16.35	13.81	11.99	10.33	39.27	33.15	33.16
11	72.69	56.06	46.42	39.95	34.47	29.97	54.44	45.79	56.93
12	45.43	35.7	28.19	23.45	18.3	15.83	48.46	40.40	57.53
13	26.49	19.64	14.06	9.92	7.48	5.7	10.84	7.15	11.7
14	141.98	112.7	89.36	69.05	51.05	35.97	152.57	128.12	164.1
15	-	8.59	5.76	3.94	3.12	2.05	5.91	3.67	7
16	-	7.64	5.68	4.2	3.55	2.84	3.78	2.66	11.82
17	42.34	31.21	27.05	23.24	19.18	16.89	33.02	28.60	26.15
Median	39.49	20.59	17	15.31	13.83	11.94	32.75	28.60	26.15
P value	0.281	0.102	0.004	0.000	0.000	0.000	0.687	0.031	-

P value, GTV_PET_ VS IGTV_10_.

### CI

**Figure 2:**
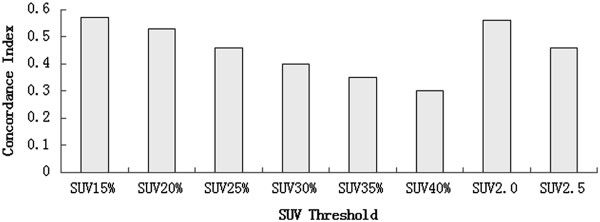
**Median concordance index of GTV**_**PET**_ **and IGTV**_**10**_ **.**

Figure 2 shows the median CIs of the PET volumes and the IGTV_10_ volumes. The median CIs ranged from 0.30 to 0.57 (F = 2.526, P = 0.018). The best fit for CI was at SUV15% (0.57), followed by SUV 2.0 (0.56). The CIs of IGTV_10_ and GTV_PET_ were inversely correlated with the centroid shifts in 3D directions (r = −0.668, −0.699, −0.728, −0.728, −0.801, −0.755, −0.711, −0.787; P = 0.007, 0.002, 0.001, 0.001, 0.000, 0.000, 0.001, 0.000).

### DI

**Figure 3:**
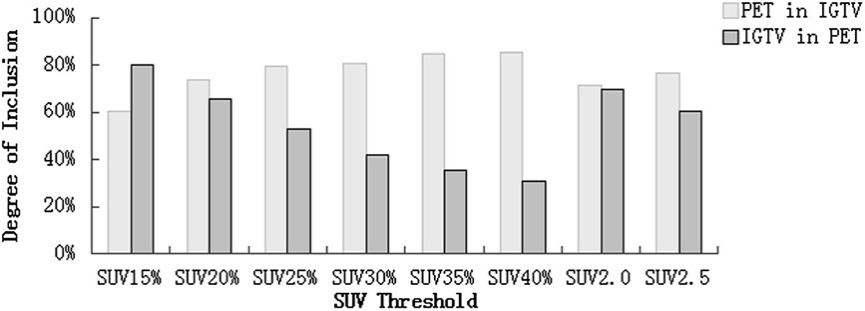
**Median degree of inclusion of GTV**_**PET**_ **in IGTV**_**10**_ **, and IGTV**_**10**_ **in GTV**_**PET**_ **.**

Figure 3 showed the median DIs of GTV_PET_ in IGTV_10_, and IGTV_10_ in GTV_PET_. The median DIs of IGTV_10_ in GTV_PET_ ranged from 0.31 to 0.80 (F = 7.814, P = 0.000). The best DI was IGTV_10_ in GTV_PET15%_. The median DIs of GTV_PET_ in IGTV_10_ ranged from 0.60 to 0.85 (F = 1.017, P = 0.422). The best DIs were GTV_PET35%_ and GTV_PET40%_ in IGTV_10_.

## Discussion

Accurate definition of the target volume, ideally incorporating metabolic information, becomes paramount importance in the current trend in NSCLC treatment planning [21]. A number of studies compared 3DCT volumes with ^18^ F-FDG PET/CT volumes of NSCLC [22–24]. However, the best methodology for applying ^18^ F-FDG PET/CT to IGTV definition is not currently well established. To the best of our knowledge, there is few study that has compared tumor sizes and CI values between GTV_PET_ and IGTV_10_ in contouring NSCLC.

In this study, we determined the IGTV_10_ from 10 phases of the 4DCT dataset and used them as the reference to find the optimal threshold that yield the best match between the GTV_PET_ and IGTV_10_ in both the target size and the spatial conformity. Our study revealed that GTV_PET_ using a threshold setting of SUV15% approximated most closely to the IGTV_10_ with the lowest median percentage volume changes. When using the threshold level of ≥ SUV25% and/or ≥ SUV2.5, the PET-based tumor sizes were estimated to be smaller than the IGTV_10_. Therefore, on the basis of the results of our study, the SUV threshold setting of ≥25% and/or ≥2.5 is not suitable for IGTV contouring in NSCLC.

Analogously, Hanna et al. [25] compared volumes from a manual method and five automated PET segmentation techniques to 4DCT-derived ITV and found that none of the PET target volumes approximated closely to the 4DCT target volumes. However, in their study, the patient’s PET/CT and 4DCT scans were not acquired on the same day or in identical position. In this circumstance, it was possible that changes in tumor geometry or size occurred and potentially increased the likelihood of a mismatch between the PET-based contours and the 4DCT-based contours [25]. Caldwell et al. [16] reported that using a threshold as low as 15% of the maximum value could account for respiratory motion and more accurately depict the true extension of the moving target. Another phantom study by Okubo et al. [26] concluded that when a threshold value of 35% of the measured maximum FDG activity was adopted, the sizes of PET delineation were almost the same for static and moving phantom spheres of 22 mm or more in the axial plane. Our study was similar to the result of Caldwell et al., but smaller than the result of Okubo et al. This is possible because patients enrolled in our study had a range of tumor sizes and positions. Moreover, the 15% threshold method was not suitable for contouring some lung tumors that have low SUV, because it might fail to distinguish tumor from background lung activity. Nevertheless, Okubo et al. suggested that the threshold of 35% of measured maximum FDG activity was only a provisional criterion for tumors of 2–4 cm given that appropriate threshold values could be changed on the basis of the tumor size [26]. In addition, it should be acknowledged that any phantom studies versus clinical comparison is limited. For example, unlike in real tumors, the FDG distribution in the spheres of the phantoms and in the background was homogenous.

Similarity in absolute volume does not mean identity in the space location. Our results indicated that GTV_PET15%_, GTV_PET20%_ and GTV_PET2.0_ showed no significant difference with IGTV_10_ in target volume. However, the CIs of them were significantly lower than 1.0. The best CI was between IGTV_10_ and GTV_PET15%_, which was only 0.57. It is not surprising as GTV_PET15%_ is the biggest volume and hence has the greatest degree of potential overlap. Based on this consideration, this may not make it the most accurate. The poor CIs suggested great unconformity between what was indicated abnormal on PET image and on CT image. One of the reasons is that shape and/or positional alterations between IGTV_10_ and GTV_PET_ had occurred. Our study showed that the minimum variation in the centroid coordinate position in the 3D direction of GTV_PET_ and IGTV_10_ was 0.38 cm (median). Moreover, the CIs were inversely correlated with the centroid shifts in 3D directions. Although patients in our study were immobilized in the same position for both 4DCT and PET/CT, millimetric precision in set-up using immobilization devices may not be feasible. Furthermore, a rigid registration might not be sufficient for lung tumors. Hence, registration error may inevitable affect the spatial position between GTV_PET_ and IGTV_10_. In addition, it is possible that some of this difference may be related to differences in the patient’s breathing pattern between acquiring the PET/CT and 4DCT. Different breathing pattern can influence tumor size, shape and distribution of activity on the free-breathing PET images [27].

Hanna et al. [25] used the Dice similarity coefficient (DSC) to assess volumetric, shape and positional similarity in the PET-generated target volumes and 4DCT target volumes. Their study revealed that the highest DSCs (mean) were 0.64. Grills et al. [21] investigated the impact of PET/CT for GTV definition in NSCLC using the matching index (similar to CI in our study) to compare the GTV, as defined by CT, with the GTV defined by PET co-registered with CT. In their study, the mean matching index was 0.65. Gondi et al. [23] demonstrated that CI values of NSCLC with the incorporation of FDG-PET and CT were 0.44. The results of our study were similar to data published in prior studies [21, 23, 25]. In addition, although CI can provide the most information on both volume change and positional change [28], it cannot quantify the percentage of one volume included by another volume. Further analyzing the inclusion relation between GTV_PET15%_ and IGTV_10_, there was 20% of (median) GTV_PET15%_ not included in IGTV_10_ and 40% of (median) IGTV_10_ not included in GTV_PET15%_. It suggested that ITGV_10_ did not encompass GTV_PET15%_ completely or vice versa. Therefore, we concurred with Gondi [21] who concluded that although the quantitative absolute target volume could sometimes be similar between CT and PET, the qualitative target locations can be significantly different.

One limitation of our study is the small number of patients studied, so that subgroup analyses were limited. Therefore, a larger cohort of patients with many different tumor sizes and locations should be conducted to further investigate the relationship of using 3D-PET/CT and 4DCT for contouring IGTV of NSCLC. We are continuing our work to enroll more patients for further clinical investigations.

## Conclusion

None of the PET based contours had both close spatial and volumetric approximation to the 4DCT IGTV_10_. At present 3D-PET/CT should not be used for IGTV generation.

## Abbreviations

NSCLC: Non-small-cell lung cancer
ICRU: International commission on radiation units
ITV: Internal target volume
4DCT: Four-dimensional computed tomography
IGTV: Internal gross target volume
GTV: Gross tumor volumes
F-FDG: ^18^ F-fluorodeoxyglucose
PET/CT: Positron emission tomography/computer tomography
3D: Three-dimensional
RPM: Real-time positioning management
SUV_max_: Maximal standardized uptake value
ROI: Region of interest
CI: Concordance index
DI: Degree of inclusion
LR: Left-right
AP: Anterior-posterior
CC: Cranial-caudal
DSC: Dice similarity coefficient.
